# Sex-based analysis of clinical outcomes in elderly patients with esophageal squamous cell carcinoma post-esophagectomy: a propensity score matching analysis

**DOI:** 10.3389/fonc.2025.1549123

**Published:** 2025-04-14

**Authors:** Kexun Li, Simiao Lu, Changding Li, Jie Mao, Huan Zhang, Kangning Wang, Guangyuan Liu, Yunchao Huang, Yongtao Han, Lin Peng, Xuefeng Leng

**Affiliations:** ^1^ Department of Thoracic Surgery, Sichuan Clinical Research Center for Cancer, Sichuan Cancer Hospital & Institute, Sichuan Cancer Center, Affiliated Cancer Hospital of University of Electronic Science and Technology of China (Sichuan Cancer Hospital), Chengdu, China; ^2^ Department of Thoracic Surgery I, Third Affiliated Hospital of Kunming Medical University (Yunnan Cancer Hospital, Yunnan Cancer Center), Kunming, China; ^3^ School of Public Health, Chongqing Medical University, Chongqing, China; ^4^ Department of Thoracic Surgery, Zigong First People’s Hospital, Sichuan, Zigong, China

**Keywords:** elderly ESCC patients, sex, overall survival, disease-free survival, postoperative complications

## Abstract

**Background:**

Esophageal squamous cell carcinoma (ESCC) is a common and aggressive form of esophageal cancer, particularly prevalent in East Asia. This study aimed to investigate the impact of sex on clinical outcomes, including survival and postoperative complications, in elderly ESCC patients following esophagectomy.

**Methods:**

We conducted a retrospective cohort study using data from the Sichuan Cancer Hospital & Institute Esophageal Cancer Case Management Database, involving patients aged 70 years and older who underwent esophagectomy from May 2016 and August 2021. Patients were grouped by sex, and subgroup analyses were performed on non-smoking, non-drinking patients. OS and DFS were analyzed using the Kaplan-Meier method, and between-group comparisons were conducted using the log-rank test. Propensity score matching (PSM) was applied to adjust for potential confounders.

**Results:**

Although females showed a longer median OS (60.2 months) compared to males (40.0 months), the difference was not statistically significant after PSM (HR = 0.885, P = 0.573). Similarly, no significant differences were observed in DFS between sexes. In non-smoking, non-drinking subgroups, OS and DFS remained higher but without significant sex-based differences. Postoperative adverse events such as pulmonary infection and anastomotic leakage were common across groups.

**Conclusions:**

While sex does not significantly affect OS and DFS in elderly ESCC patients, male patients may experience higher rates of certain postoperative complications, such as abnormal liver function and pneumothorax.

## 1 Introduction

Esophageal squamous cell carcinoma (ESCC) is one of the most prevalent and aggressive forms of esophageal cancer (EC), particularly in regions such as East Asia, where its incidence is notably high (1–4). Surgical resection, specifically esophagectomy, remains the cornerstone of treatment for resectable ESCC, with the primary aim of removing the tumor and affected portions of the esophagus. However, despite advancements in surgical techniques and perioperative care, the prognosis for patients undergoing esophagectomy remains suboptimal (5–8). In recent years, the treatment of ESCC has evolved into a multimodal approach, combining surgery with neoadjuvant therapies such as radiotherapy, chemotherapy, and, more recently, immunotherapy, particularly for patients with locally advanced disease. Neoadjuvant treatments have been shown to reduce tumor burden, increase the likelihood of complete resection, and improve overall survival (9–13). Furthermore, post-operative adjuvant therapies, tailored according to pathological findings, play a critical role in reducing recurrence rates and enhancing long-term outcomes (5, 6, 14, 15).

According to the Global Burden of Disease Study, while the overall incidence of EC has demonstrated a decreasing trend globally in recent years, the aging population has resulted in an increased number of elderly patients diagnosed with the disease. As the elderly population continues to expand, the absolute number of older adults affected by EC is rising, even as age-specific incidence rates decline (16–18). This demographic shift underscores the necessity for more targeted research on elderly ESCC patients, who may encounter unique challenges due to age-related physiological changes, comorbidities, and potential treatment-related complications.

While a range of demographic and biological factors may influence treatment outcomes for ESCC, sex has recently garnered increasing attention as a potential determinant of prognosis. Studies across various cancer types have indicated that biological sex may affect tumor biology, treatment response, and overall survival (18). In the context of EC, males exhibit a significantly higher incidence than females. This disparity is partly attributed to lifestyle factors, as male patients are more likely to engage in behaviors such as smoking, excessive alcohol consumption, and irregular sleep patterns (19–21). Furthermore, males often encounter greater social, economic, and familial pressures, which may adversely impact their health and recovery. Research has suggested that male ESCC patients tend to experience worse survival outcomes compared to their female counterparts; however, the role of sex in influencing post-surgical complications remains underexplored (22).

Given the sex-related differences in lifestyle and health outcomes, it is essential to investigate not only the overall survival and complication rates between male and female ESCC patients but also to examine these differences in subgroups of patients who do not smoke or consume alcohol. To address these gaps, we conducted a retrospective cohort study to explore the impact of sex on clinical outcomes, including survival and postoperative complications, in elderly ESCC patients following esophagectomy. Additionally, we extended our analysis to non-smoking, non-drinking patients to better understand whether sex differences persist in the absence of these lifestyle factors.

## 2 Materials and methods

### 2.1 Study design and patient selection

**Figure 1:**
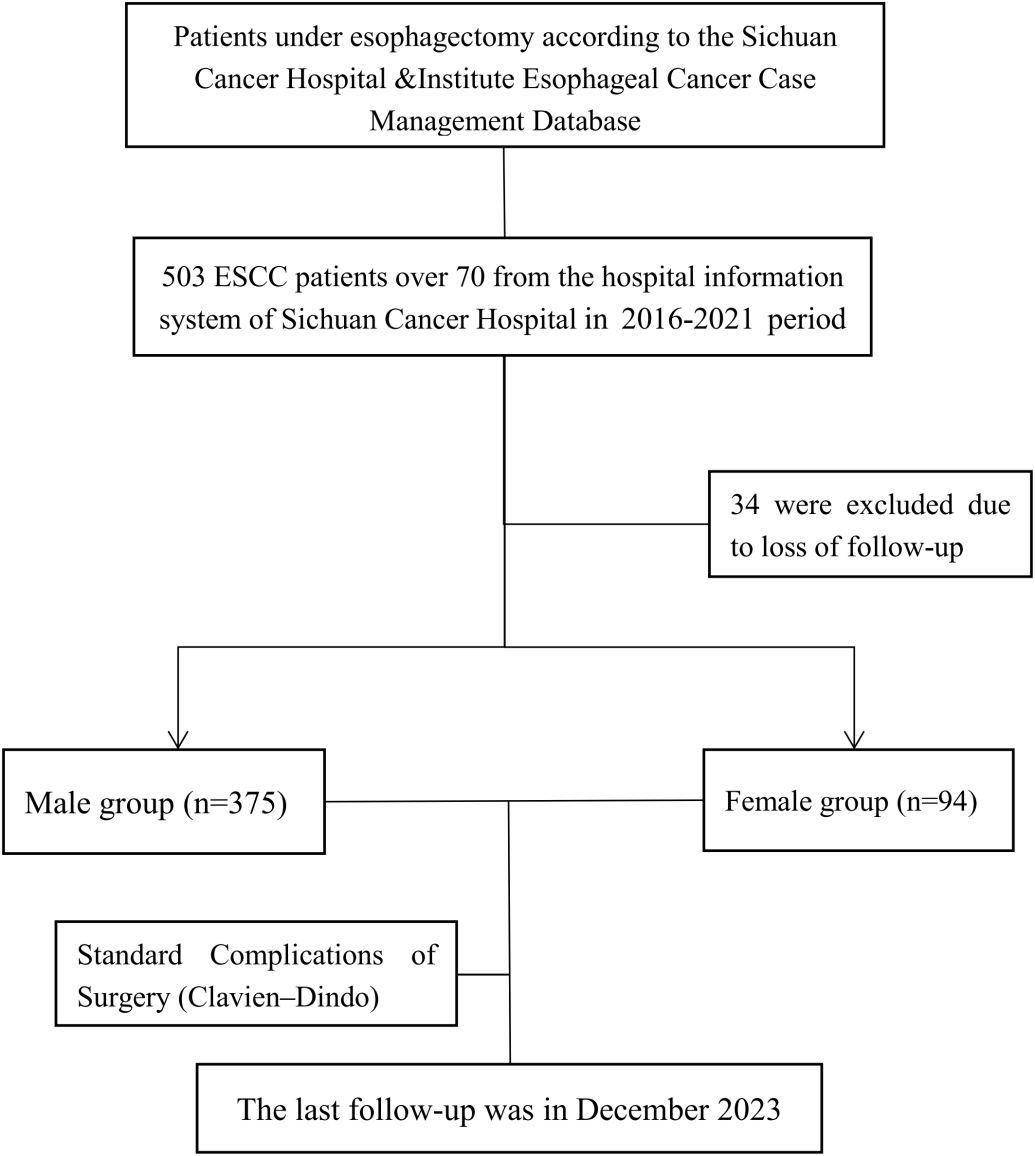
CONSORT diagram of patient selection.

This retrospective cohort study was conducted using a prospectively maintained Sichuan Cancer Hospital & Institute Esophageal Cancer Case Management Database (SCCH-ECCM Database) from the Sichuan Cancer Hospital & Institute. We included patients diagnosed with ESCC who underwent esophagectomy from May 2016 and August 2021. The study exclusively focused on elderly patients aged 70 years and older. Inclusion criteria consisted of histologically confirmed squamous cell carcinoma located in the thoracic esophagus with no evidence of distant metastasis, as determined by clinical and imaging assessments. Patients were excluded if they had non-thoracic esophageal tumors, non-squamous cell carcinoma pathology, or incomplete clinical data, as shown in **Figure 1**. Follow-up data were collected until the final cut-off date on December 20, 2023.

### 2.2 Grouping and outcome measures

Patients were categorized into groups based on sex (male vs. female), and additional subgroup analyses were conducted to evaluate differences in clinical outcomes between non-smoking, non-drinking males and females. The primary outcomes of interest included overall survival (OS) and disease-free survival (DFS). OS was defined as the duration from the date of treatment to the date of death from any cause or until the last follow-up (December 20, 2023). DFS was defined as the time from the date of treatment to the date of either documented disease recurrence (local or distant relapse), death from any cause, or the last follow-up (December 20, 2023), whichever occurs first. All tumor staging was performed in accordance with the 8th edition of the TNM classification established by the Union for International Cancer Control (UICC) and the American Joint Committee on Cancer (AJCC) guidelines (5). Pathological diagnoses were reviewed by two independent pathologists, with a third expert consulted in instances of discrepancy.

### 2.3 Criteria and characteristics of the adverse events

Postoperative adverse events were categorized based on severity using the Clavien-Dindo classification system. Complications were defined as any deviation from the normal postoperative course, and were graded accordingly (23–25). Additionally, patients’ body mass index (BMI) was recorded and classified into three groups according to the World Health Organization (WHO) standards, to account for potential differences in body composition that could influence surgical outcomes. All adverse events were reviewed and verified by the surgical team members for accuracy (26, 27).

### 2.4 Statistical analyses

Descriptive statistics were used to summarize baseline demographic and clinical characteristics. Continuous variables are presented as means with standard deviations (SD), while categorical variables are shown as frequencies and percentages. Survival outcomes, including OS and DFS, were estimated using the Kaplan-Meier method, and between-group comparisons were performed using the log-rank test. Univariate Cox proportional hazards models were used to examine potential prognostic factors for OS and DFS, including age, sex, tumor stage, and karnofsky performance status (KPS) scores. Significant variables from the univariate analysis (p < 0.05) were entered into a multivariate Cox regression model to identify independent risk factors. Additionally, we conducted a propensity score matching (PSM) analysis to compare outcomes between males and females, as well as between patients with and without smoking or drinking habits. Matching was performed using a nearest neighbor algorithm without replacement, based on a logistic regression model incorporating variables such as age, tumor stage, and KPS scores to ensure balanced comparison groups. All statistical analyses were carried out using RStudio version 4.3.0. A two-sided p-value of less than 0.05 was considered statistically significant.

### 2.5 Ethical considerations

This study was conducted in compliance with ethical standards and received approval from the Ethics Committee for Medical Research and New Medical Technology of Sichuan Cancer Hospital (Approval No. SCCHEC-02-2024-191). Informed consent was waived given the retrospective nature of the study, and all procedures adhered to the principles outlined in the Declaration of Helsinki (2013 revision). The authors affirm accountability for all aspects of the research to ensure the integrity and accuracy of the findings.

## 3 Results

### 3.1 Demographic data

A total of 469 elderly patients aged 70 years and above who underwent esophagectomy for ESCC were included in this study. The cohort was divided into two groups based on sex: 375 males (80.0%) were categorized into the male group and 94 females (20.0%) were categorized into the female group. Among these, 27 patients (5.8%) were aged 80 years or older, while the majority, 442 patients (94.2%), were aged between 70 and 79 years. Smoking and alcohol consumption were prevalent among the cohort, with 241 patients (51.4%) reporting a history of smoking and 245 patients (52.2%) reporting alcohol consumption. Preoperative functional status, as assessed by the KPS, revealed that 350 patients (74.6%) had a KPS score of 90-100, indicating a good functional status, while 119 patients (25.4%) had a KPS score below 90, reflecting a reduced preoperative functional capacity. In terms of BMI, 34 patients (7.2%) were classified in the Low-BMI category (<18.5 kg/m²), 360 patients (76.8%) in the Normal-BMI group (18.5-24.9 kg/m²), and 75 patients (16.0%) in the High-BMI group (≥25 kg/m²). Clinical TNM staging (cTNM) prior to surgery showed that 342 patients (72.9%) were diagnosed with locally advanced disease (stage III or above), and 72 patients (15.4%) received neoadjuvant therapy. Postoperative pathological TNM staging (pTNM) revealed that 227 patients (48.4%) were diagnosed with stage III or higher after surgery, indicating a predominance of advanced tumors in the cohort. In the subgroup of patients who neither smoked nor consumed alcohol (n = 187), the distribution between the sexes was more balanced compared to the overall cohort. 100 patients (53.5%) were categorized into the male group, while 87 patients (46.5%) were categorized into the female group. The pathological staging in this group also indicated a significant proportion of advanced disease, with 77 patients (41.2%) having stage III or higher tumors. A total of 27 patients (14.4%) had undergone neoadjuvant therapy prior to surgery (**Table 1** and **Table 2**).

**Table 1:** Demographic characteristics of the 2 groups.

Characteristic		Before PSM		*P* value	After PSM		*P* value
	Total (n=469)	Male (n=375)	Female (n=94)		Male (n=94)	Female (n=94)	
Smoking				<0.001			
Yes	241	238 (63.47%)	3 (3.19%)		4 (4.26%)	3 (3.19%)	0.700
No	228	137 (57.56%)	91 (96.81%)		90 (95.74%)	91 (96.81%)	
Alcohol				<0.001			0.419
Yes	245	239 (63.73%)	6 (6.38%)		9 (9.57%)	6 (6.38%)	
No	224	136 (36.27%)	88 (93.62%)		85 (90.43%)	88 (93.62%)	
Age, years				0.485			1.000
median (range)	73 (70–88)	73 (70–85)	73 (70–88)		73 (70–88)	73 (70–88)	
<80	442	352 (93.87%)	90 (95.74%)		90 (95.74%)	90 (95.74%)	
≥80	27	23(6.13%)	4 (4.26%)		4 (4.26%)	4 (4.26%)	
BMI				0.090			0.658
Low	34	28 (7.47%)	6 (6.38%)		7 (7.45%)	6 (6.38%)	
Normal	360	294 (78.4%)	66 (70.21%)		70 (74.47%)	66 (70.21%)	
High	75	53 (14.13%)	22 (23.40%)		17 (18.09%)	22 (23.40%)	
KPS score				0.404			0.744
≤80	119	92 (24.53%)	27 (28.72%)		25 (26.60%)	27 (28.72%)	
≥90	350	283 (75.47%)	67 (71.28%)		69 (73.40%)	67 (71.28%)	
Surgical approach				0.029			0.744
McKeown	407	319 (85.07%)	88 (93.62%)		87 (92.55%)	88 (93.62%)	
Lovr-Lewis	62	56 (14.93%)	6 (6.38%)		7 (7.45%)	6 (6.38%)	
Clinical T stage				0.391			0.984
T1	29	20 (5.33%)	9 (9.57%)		8 (8.51%)	9 (9.57%)	
T2	67	52 (13.87%)	15 (15.96%)		16 (17.02%)	15 (15.96%)	
T3	324	262 (69.87%)	62 (65.96%)		61 (64.89%)	62 (65.96%)	
T4	49	41 (10.93%)	8 (8.51%)		9 (9.57%)	8 (8.51%)	
Clinical N stage				0.584			0.972
N0	81	64 (17.07%)	17 (18.09%)		18 (19.15%)	17 (18.09%)	
N1	282	223 (59.47%)	59 (62.77%)		59 (62.77%)	59 (62.77%)	
N2	100	82 (21.87%)	18 (19.15%)		17 (18.09%)	18 (19.15%)	
N3	6	6 (1.60%)	0 (0.00%)		0 (0.00%)	0 (0.00%)	
Clinical 8th TNM Stage				0.216			0.985
I	27	18 (4.80%)	9 (9.57%)		8 (8.51%)	9 (9.57%)	
II	100	82 (21.87%)	18 (19.15%)		19 (20.21%)	18 (19.15%)	
III	286	227 (60.53%)	59 (62.77%)		58 (61.70%)	59 (62.77%)	
IV	56	48 (12.80%)	8 (8.51%)		9 (9.57%)	8 (8.51%)	
Tumor location				<0.001			0.057
Upper	61	38 (10.13%)	23 (24.47%)		13 (13.83%)	23 (24.47%)	
Middle	207	159 (42.40%)	48 (51.06%)		45 (47.87%)	48 (51.06%)	
Lower	201	178 (47.47%)	23 (24.47%)		36 (38.30%)	23 (24.47%)	
Neoadjuvant therapy				0.272			0.398
Yes	72	61 (16.27%)	11 (11.70%)		15 (15.96%)	11 (11.70%)	
No	397	314 (83.73%)	83 (88.30%)		79 (84.04%)	83 (88.30%)	
Thoracic surgery				0.090			1.000
MIE	111	95 (25.33%)	16 (17.02%)		16 (17.02%)	16 (17.02%)	
OE	358	280 (74.67%)	78 (82.98%)		78 (82.98%)	78 (82.98%)	

CT, chemotherapy; CRT, chemoradiotherapy; MIE, minimally invasive esophagectomy; OE, open esophagectomy; PSM, propensity score matching; BMI, body mass index; TNM, tumor, node, metastasis.

**Table 2:** Details after esophagectomy in 2 groups.

Characteristic		Before PSM		*P* value	After PSM		*P* value
	Total (n=469)	Male (n=375)	Female (n=94)		Male (n=94)	Female (n=94)	
Pathological differentiation grade				0.570			0.747
Moderate or Well G1-2	328	260 (69.33%)	68 (72.34%)		66 (70.21%)	68 (72.34%)	
Poor or undifferentiated G3-4	141	115 (30.67%)	26 (27.66%)		28 (29.79%)	26 (27.66%)	
Lymphovascular invasion				0.349			0.750
Yes	164	135 (36.00%)	29 (30.85%)		27 (28.72%)	29 (30.85%)	
No	305	240 (64.00%)	65 (69.15%)		67 (71.28%)	65 (69.15%)	
Nerve invasion				0.502			0.548
Yes	204	166 (44.27%)	38 (40.43%)		34 (36.17%)	38 (40.43%)	
No	265	209 (55.73%)	56 (59.57%)		60 (63.83%)	56 (59.57%)	
Complete resection				0.137			0.316
R0	452	359 (95.73%)	93 (98.94%)		94 (100.00%)	93 (98.94%)	
R1/R2	17	16 (4.27%)	1(1.06%)		0 (0.00%)	1(1.06%)	
Pathological T stage				0.378			0.931
T0	12	10 (2.67%)	2 (2.13%)		3 (3.19%)	2 (2.13%)	
T1	73	55 (14.67%)	18 (19.15%)		22 (23.40%)	18 (19.15%)	
T2	94	71 (18.93%)	23 (24.47%)		20 (21.28%)	23 (24.47%)	
T3	269	220 (58.67%)	49 (52.13%)		47 (50.00%)	49 (52.13%)	
T4	21	19 (5.07%)	2 (2.13%)		2 (2.13%)	2 (2.13%)	
Pathological N stage				0.283			0.848
N0	244	189 (50.40%)	55 (58.51%)		56 (59.57%)	55 (58.51%)	
N1	135	108 (28.80%)	27 (28.73%)		25 (26.60%)	27 (28.73%)	
N2	68	58 (15.47%)	10 (10.64%)		9 (9.57%)	10 (10.64%)	
N3	22	20 (5.33%)	2 (2.13%)		4 (4.26%)	2 (2.13%)	
Pathological 8th TNM Stage				0.149			0.865
I	88	65 (17.33%)	23 (24.47%)		25 (26.60%)	23 (24.47%)	
II	154	121 (32.27%)	33 (35.11%)		32 (34.04%)	33 (35.11%)	
III	193	158 (42.13%)	35 (37.23%)		32 (34.04%)	35 (37.23%)	
IV	34	31 (8.27%)	3 (3.19%)		5 (5.32%)	3 (3.19%)	

TNM, tumor, node, metastasis.

### 3.2 Overall survival and disease free survival

**Figure 2:**
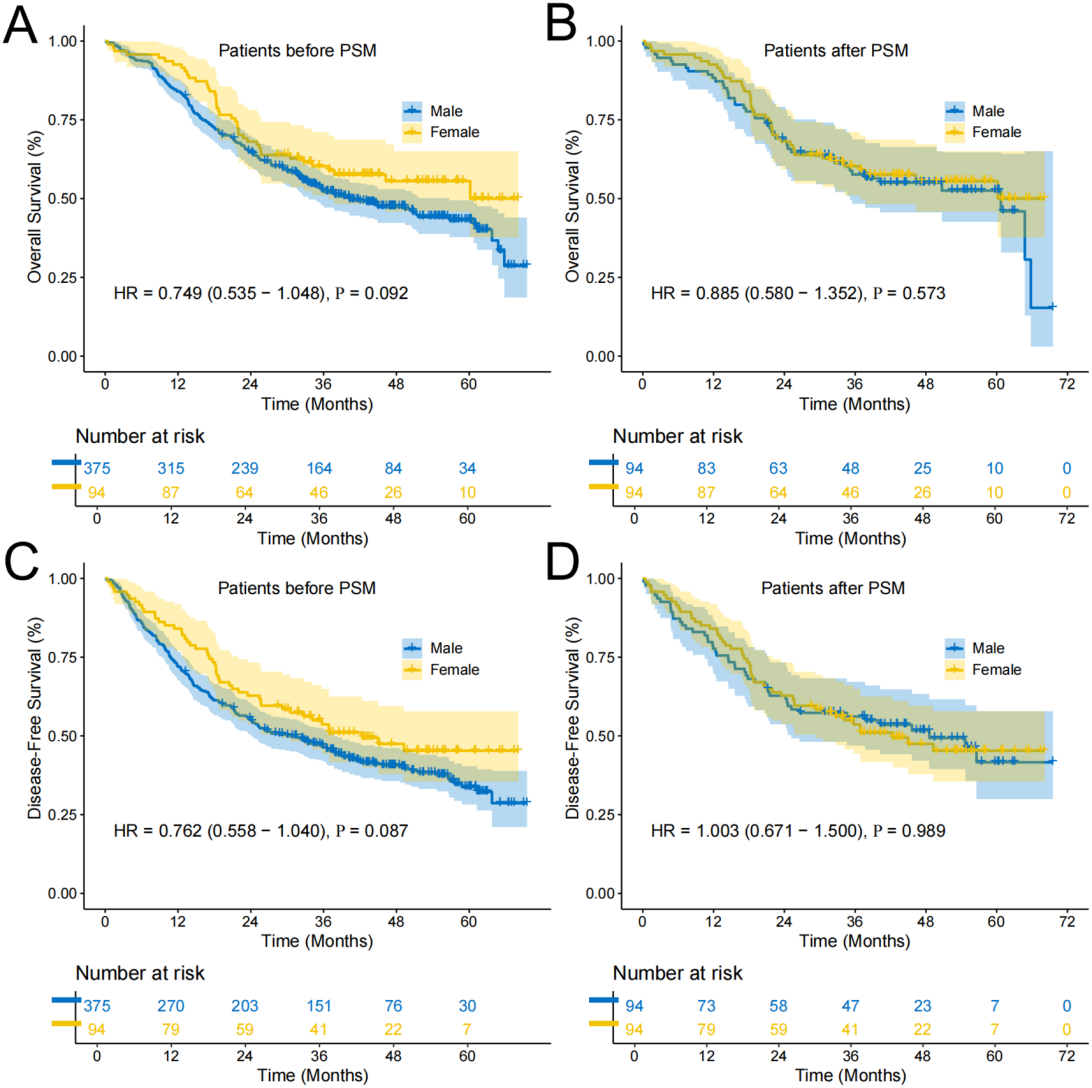
Survival curves of participants in Female and Male groups. **(A)** Overall survival curve of Female and Male groups before PSM; **(B)** Overall survival curve of Female and Male groups after PSM; **(C)** Disease-free survival curve of Female and Male groups before PSM; **(D)** Disease-free survival curve of Female and Male groups after PSM.

In the cohort of 469 elderly patients undergoing esophagectomy for ESCC, the median follow-up duration was 47.5 months. The OS analysis revealed a median OS of 51.6 months (95% CI: 38.10-65.10). When comparing survival outcomes between sexes, the median OS for male patients was 40.0 months (95% CI: 31.23-49.19), while female patients had a longer median OS of 60.2 months. Despite this difference, the survival rates at 1, 3, and 5 years were 84%, 52%, and 42% for males, and 93%, 60%, and 55% for females, respectively. However, the unadjusted HR of 0.749 (95% CI: 0.535-1.048; P=0.092) suggested no statistically significant difference in OS between the two groups (**Figure 2A**). Further analysis using 1:1 propensity score matching (PSM) confirmed the lack of significant OS difference, yielding an HR of 0.885 (95% CI: 0.580-1.352; P=0.573; **Figure 2B**). For DFS, the median DFS time was 33.0 months (95% CI: 26.84-39.16) in patients. Males experienced a median DFS of 30.4 months (95% CI: 23.67-37.13), compared to 42.3 months for females. The 1-, 3-, and 5-year DFS rates were 72%, 47%, and 34% for males, and 84%, 53%, and 44% for females, respectively. The unadjusted HR for DFS was 0.762 (95% CI: 0.558-1.040; P=0.087), indicating no significant difference (**Figure 2C**). Post-PSM analysis further supported these findings, with an HR of 1.003 (95% CI: 0.671-1.500; P=0.989; **Figure 2D**).

**Figure 3:**
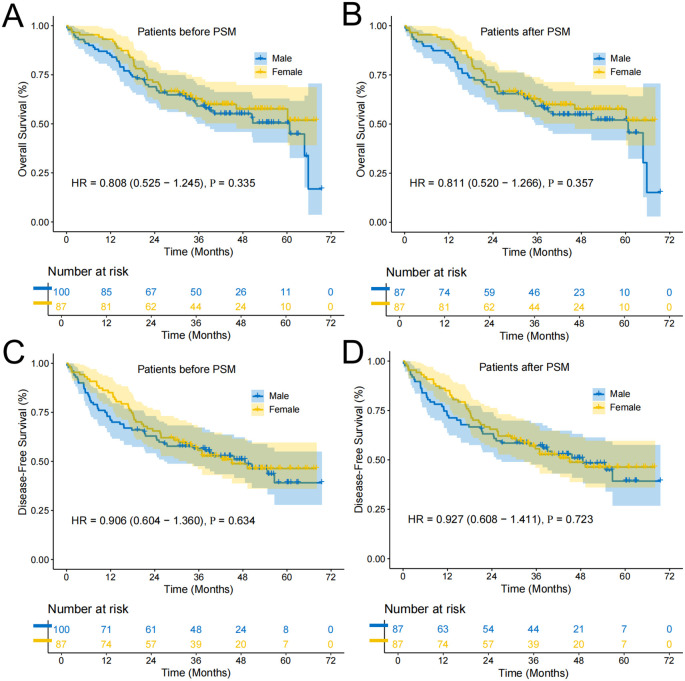
Survival curves of the subgroup of patients who neither smoked nor consumed alcohol. **(A)** Overall survival curve of Female and Male groups before PSM; **(B)** Overall survival curve of Female and Male groups after PSM; **(C)** Disease-free survival curve of Female and Male groups before PSM; **(D)** Disease-free survival curve of Female and Male groups after PSM.

In the subgroup analysis focusing on patients who neither smoked nor consumed alcohol, the median OS was notably higher at 60.72 months (95% CI: 49.38-72.06). Within this subgroup, the OS rates at 1, 3, and 5 years were 85%, 59%, and 49% for males, and 93%, 62%, and 57% for females, respectively. The unadjusted HR for OS was 0.808 (95% CI: 0.525-1.245; P=0.335), suggesting no significant survival advantage (**Figure 3A**). After applying PSM, no significant difference persisted, with an HR of 0.811 (95% CI: 0.520-1.266; P=0.357; **Figure 3B**). For DFS within this subgroup, the median time was 45.63 months (95% CI: 33.06-58.20). The DFS rates for males at 1, 3, and 5 years were 71%, 56%, and 39%, compared to 85%, 55%, and 45% for females. The unadjusted DFS HR was 0.906 (95% CI: 0.604-1.360; P=0.634), indicating no significant difference (**Figure 3C**). PSM analysis confirmed this finding, with an HR of 0.927 (95% CI: 0.608-1.411; P=0.723; **Figure 3D**).

### 3.3 Adverse events (Clavien-Dindo, 2009)

Analysis of postoperative complications and mortality showed no significant differences between male and female patients. The 30-day mortality rates were comparable between the two groups (male: 0.53% vs. female: 1.06%, P=0.999), and similarly, the 90-day mortality rates showed no statistical difference (male: 2.93% vs. female: 3.19%, P=0.999) (**Table 2**). In examining postoperative adverse events among elderly patients undergoing esophagectomy for ESCC, both male and female patients most frequently experienced complications such as pulmonary infection, anastomotic stenosis, anastomotic leakage, and hydrothorax (**Table 3**).

**Table 3:** Adverse events (Clavien–Dindo≥III, 2009).

Adverse events	Before PSM						After PSM					
	Male (n=375)			Female (n=94)			Male (n=94)			Female (n=94)		
	III	IV	V	III	IV	V	III	IV	V	III	IV	V
Anastomotic stenosis	55(14.67)			16(17.02)	1(1.06)		14(14.89)			16(9.43)	1(1.06)	
Anastomotic leakage	36(9.60)	16(4.27)	1(0.27)	7(7.47)	4(4.26)		13(13.83)	4(4.26)	1(1.06)	7(7.47)	4(4.26)	
Pulmonary infection	46(12.27)	39(10.40)	2(0.53)	5(5.32)	10(10.64)		8(8.51)	10(10.64)	2(2.13)	5(5.32)	10(10.64)	
Hydrothorax	65(17.33)			16(17.02)			16(17.02)			16(17.02)		
Respiratory failure	2(0.53)	31(8.27)	2(0.53)	1(1.06)	6(6.38)		2(2.13)	5(5.32)	2(2.13)	1(1.06)	6(6.38)	
Heart failure	15(4.00)	7(1.87)	1(0.27)	2(2.13)	1(1.06)		4(4.26)	2(2.13)	1(1.06)	2(2.13)	1(1.06)	
Postoperative hoarseness	14(3.73)	2(0.53)		4(4.26)	1(1.06)		2(2.13)			4(4.26)	1(1.06)	
Postoperative bleeding	13(3.47)	5(1.33)		4(4.26)	2(2.13)		1(1.06)	3(3.19)		4(4.26)	2(2.13)	
Arrhythmia	19(5.07)	4(1.07)		2(2.13)	1(1.06)		2(2.13)	2(2.13)		2(2.13)	1(1.06)	
Pneumothorax	28(7.47)			2(2.13)			12(12.77)			2(2.13)		
Abnormal liver function	13(3.47)	1(0.27)			1(1.06)		7(7.47)				1(1.06)	
Fever	14(3.73)			2(2.13)			4(4.26)			2(2.13)		
Pulmonary atelectasis	9(2.40)			3(3.19)			2(2.13)			3(3.19)		
Suspected anastomotic leakage	2(0.53)			1(1.06)						1(1.06)		
Chylous fistula	6(1.60)	5(1.33)		1(1.06)			1(1.06)	2(2.13)		1(1.06)		
ARDS		5(1.33)			2(2.13)			2(2.13)			2(2.13)	
Pyothoraxs	2(0.53)	2(0.53)						2(2.13)				
Wound infection	2(0.53)	1(0.27)		1(1.06)	1(1.06)		1(1.06)			1(1.06)	1(1.06)	
Pulmonary embolism	1(0.27)	1(0.27)	1(0.27)						1(1.06)			
Delirium	1(0.27)	1(0.27)										
Thrombosis	5(1.33)			1(1.06)			1(1.06)			1(1.06)		
Ketosis		1(0.27)										
Renal injury	4(1.07)	1(0.27)		1(1.06)	1(1.06)		1(1.06)			1(1.06)	1(1.06)	
Tracheal injury			1(0.27)		2(2.13)				1(1.06)		2(2.13)	
Cerebral infarction				1(1.06)						1(1.06)		
Gastric perforation		1(0.27)						1(1.06)				
Diaphragmatic hernia		1(0.27)										

**Figure 4:**
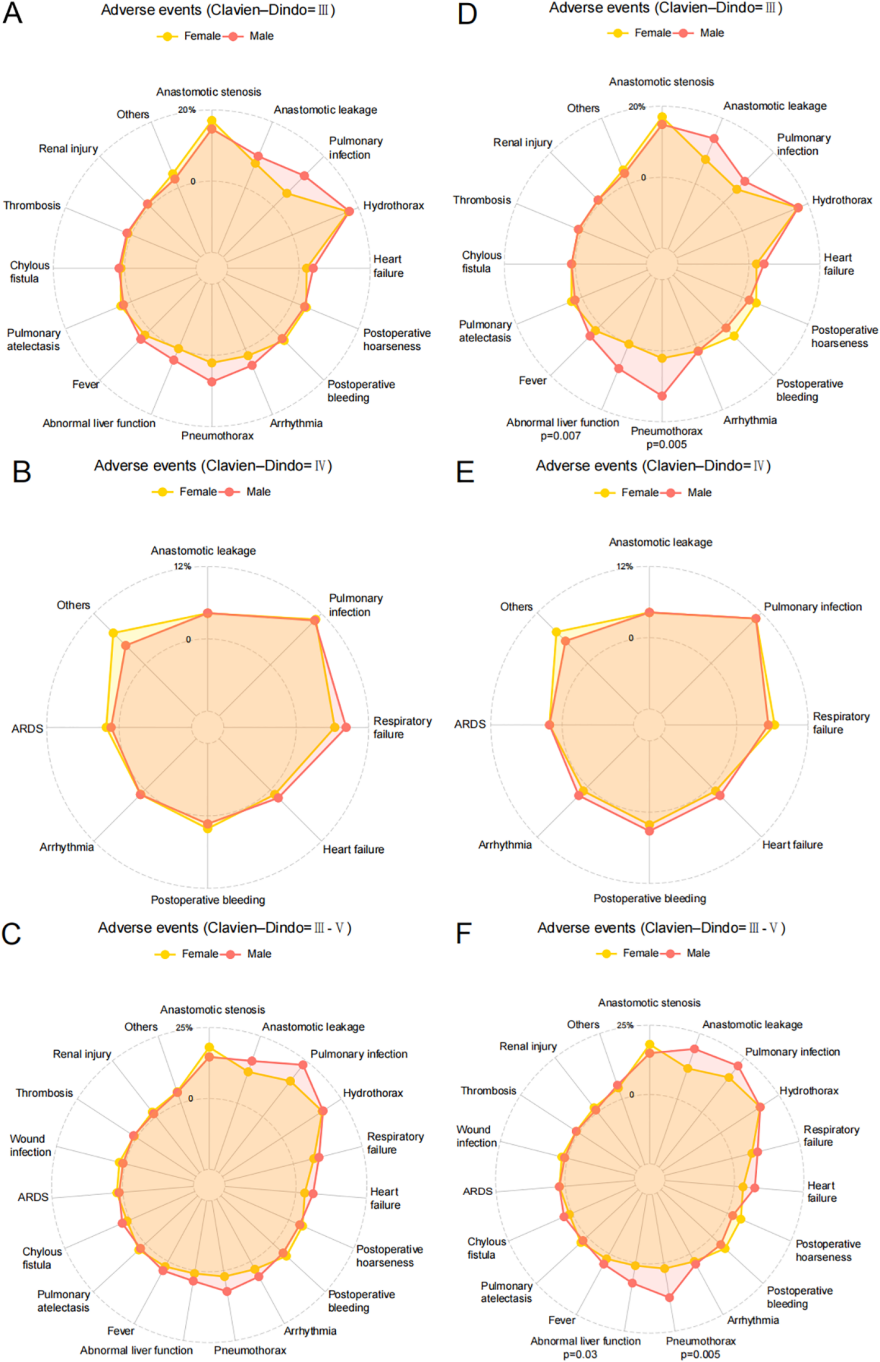
Adverse events of participants in Female and Male groups. **(A)** Clavien-Dindo grade III complications of Female and Male groups before PSM; **(B)** Clavien-Dindo grade IV complications of Female and Male groups before PSM; **(C)** Clavien-Dindo grade III-V complications of Female and Male groups before PSM; **(D)** Clavien-Dindo grade III complications of Female and Male groups after PSM; **(E)** Clavien-Dindo grade IV complications of Female and Male groups after PSM; **(F)** Clavien-Dindo grade III-V complications of Female and Male groups after PSM.

Prior to PSM, there were no statistically significant differences between male and female patients in the incidence of severe complications (Clavien-Dindo grade III or higher) across various categories (**Figure 4**). However, after PSM, which accounts for potential confounding variables and balances the comparison groups, specific differences in complication rates emerged. Notably, male patients demonstrated a significantly higher incidence of abnormal liver function compared to female patients (P=0.03), indicating a potential sex-related vulnerability in hepatic response or recovery post-surgery. Additionally, the occurrence of pneumothorax was significantly greater in male patients than in females (P=0.005) (**Figure 4**).

**Figure 5:**
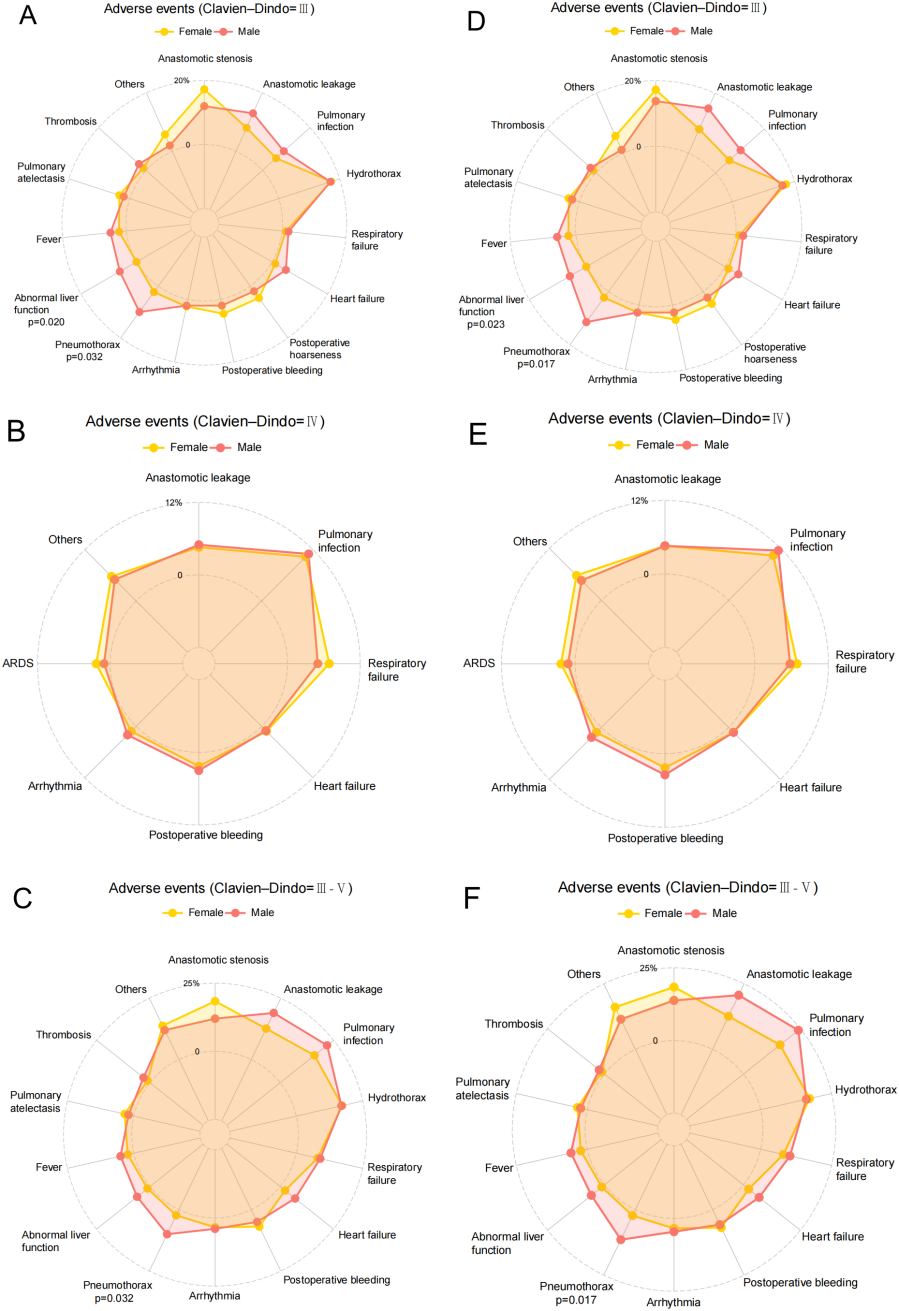
Adverse events of the subgroup of patients who neither smoked nor consumed alcohol. **(A)** Clavien-Dindo grade III complications of Female and Male groups before PSM; **(B)** Clavien-Dindo grade IV complications of Female and Male groups before PSM; **(C)** Clavien-Dindo grade III-V complications of Female and Male groups before PSM; **(D)** Clavien-Dindo grade III complications of Female and Male groups after PSM; **(E)** Clavien-Dindo grade IV complications of Female and Male groups after PSM; **(F)** Clavien-Dindo grade III-V complications of Female and Male groups after PSM.

In further subgroup analysis focusing on patients who neither smoked nor consumed alcohol, additional insights into postoperative complications emerged. Prior to PSM, among Clavien-Dindo grade III complications, male patients showed significantly higher rates of abnormal liver function (P=0.020) and pneumothorax (P=0.032) compared to female patients (**Figure 5A**). However, for Clavien-Dindo grade IV complications, no statistically significant differences were observed between male and female patients (**Figure 5B**). When considering Clavien-Dindo grade III or higher complications in this subgroup, male patients exhibited a significantly higher incidence of pneumothorax (P=0.032) compared to females, while other complications showed no significant differences between sexes (**Figure 5C**). After applying PSM to balance potential confounding factors, the analysis revealed persistent differences. For Clavien-Dindo grade III complications, male patients continued to show significantly higher rates of abnormal liver function (P=0.023) and pneumothorax (P=0.017) compared to female patients (**Figure 5D**). Similarly, for Clavien-Dindo grade III or higher complications, the significant difference in pneumothorax incidence (P=0.017) between male and female patients remained (**Figure 5F**).

### 3.4 Risk factors

**Figure 6:**
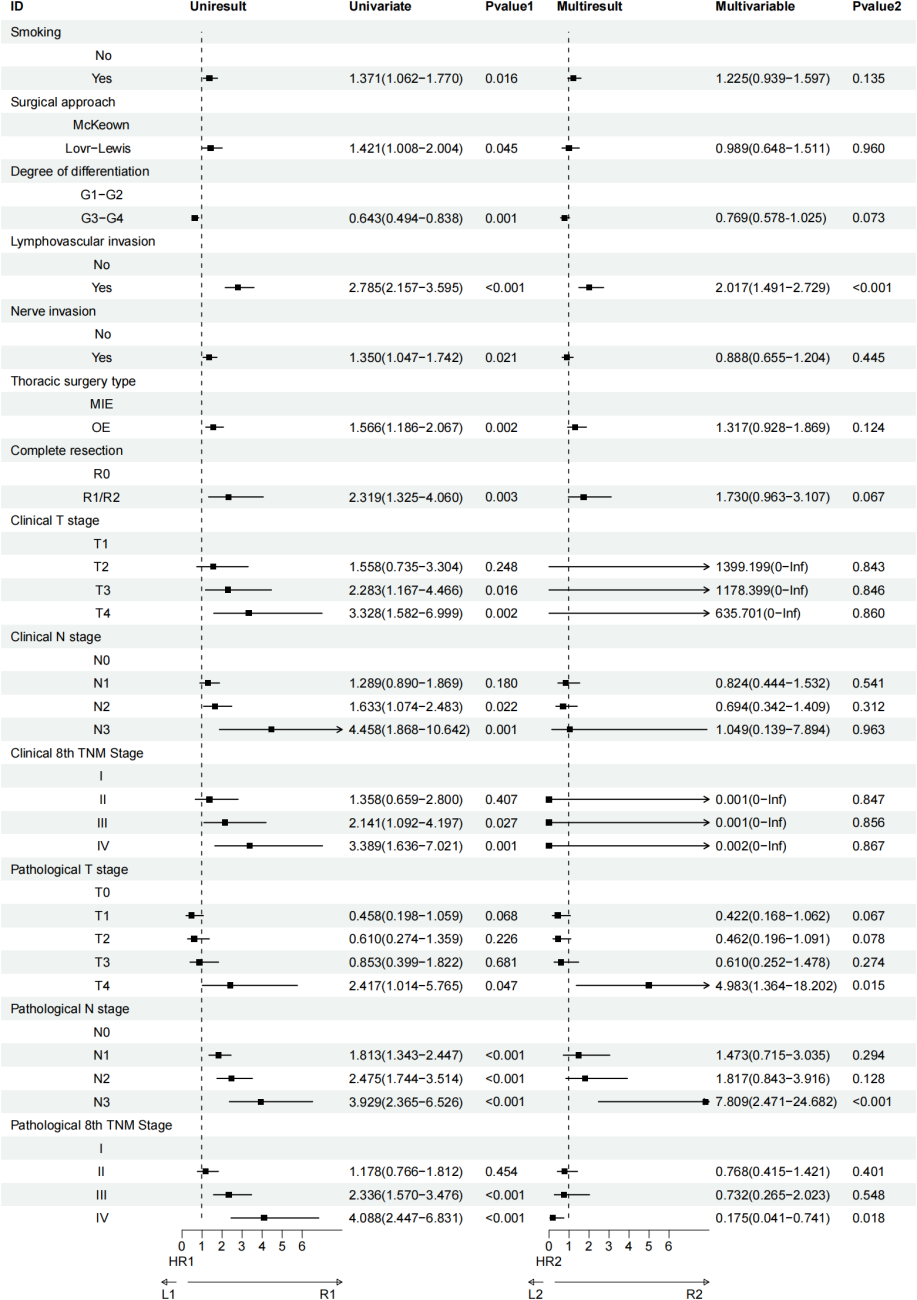
Univariate and multivariate Cox regression analyses regarding factors affecting OS of patients.

**Figure 7:**
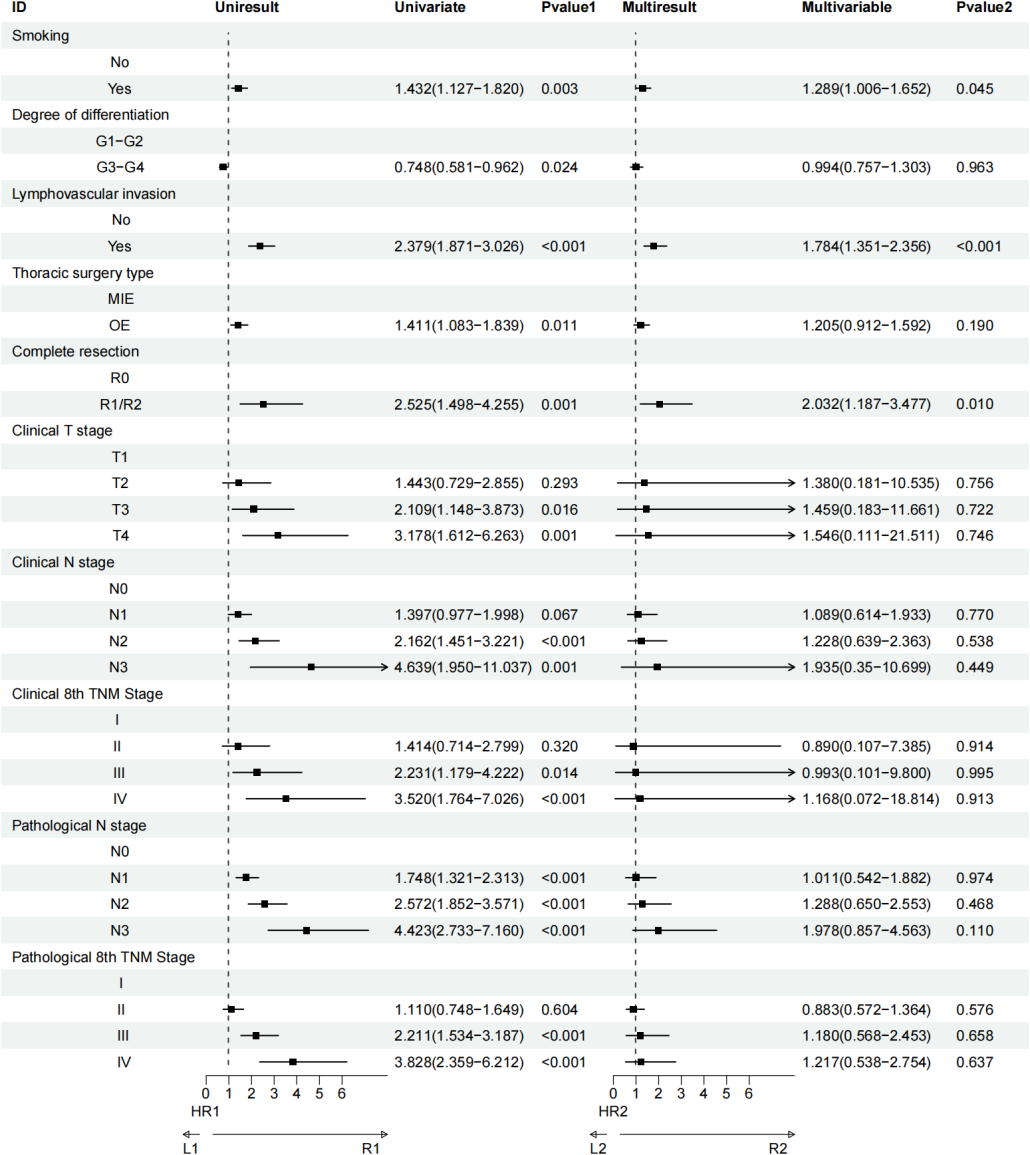
Univariate and multivariate Cox regression analyses regarding factors affecting DFS of patients.

Univariate analysis identified several factors associated with OS in elderly patients with ESCC following esophagectomy. These factors included smoking status, surgical approach, degree of tumor differentiation, lymphovascular invasion, nerve invasion, type of thoracic surgery, completeness of resection, clinical T stage (cT), clinical N stage (cN), clinical TNM stage (cTNM), pathological T stage (pT), pathological N stage (pN), and pathological TNM stage (pTNM). When these variables were further examined through multivariate Cox regression analysis, four factors emerged as independently significant predictors of OS. Lymphovascular invasion showed the strongest impact, with a statistically significant association (HR=2.017, 95% CI:1.491−2.729; P < 0.001), followed by pathological T4 stage (HR=4.983, 95% CI:1.364−18.202; P = 0.015), pathological N3 stage (HR=7.809, 95% CI:2.471−24.682; P < 0.001), and pathological stage IV (HR=0.175, 95% CI:0.041−0.741; P = 0.018) (**Figure 6**). For disease-free survival (DFS), univariate analysis highlighted smoking, degree of differentiation, lymphovascular invasion, type of thoracic surgery, completeness of resection, clinical T stage, clinical N stage, clinical TNM stage, pathological N stage, and pathological TNM stage as factors impacting DFS. Subsequent multivariate analysis revealed three variables with a significant association with DFS: smoking (HR=1.289, 95% CI:1.006−1.652; P = 0.045), lymphovascular invasion (HR=1.784, 95% CI:1.351−2.356; P < 0.001), and completeness of resection (HR=2.032, 95% CI:1.187−3.477; P = 0.010) (**Figure 7**).

## 4 Discussion

This comprehensive retrospective study investigated sex-based differences in clinical outcomes among elderly patients undergoing esophagectomy for ESCC. The analysis of survival outcomes demonstrated that while female patients tended to have longer median OS (60.2 months vs 40.0 months) and DFS (42.3 months vs 30.4 months) compared to male patients, these differences did not reach statistical significance, both before and after PSM. This finding suggests that biological sex alone may not be a decisive factor in determining survival outcomes in elderly ESCC patients undergoing surgical treatment. Notably, our study revealed distinct patterns in postoperative complications between male and female patients. After PSM, male patients showed significantly higher rates of abnormal liver function and pneumothorax. These differences persisted even in the subgroup of non-smoking, non-drinking patients, suggesting that these complications may be influenced by intrinsic sex-based biological differences rather than lifestyle factors alone.

Our findings regarding higher complication rates in male patients, particularly liver dysfunction and pneumothorax, have important clinical implications. These observations suggest the need for sex-specific approaches to perioperative care and risk assessment. Healthcare providers might consider implementing more intensive monitoring and preventive measures for male patients who may be at higher risk for these specific complications. The persistence of these sex-based differences in complications among non-smoking, non-drinking patients is particularly intriguing. This suggests that underlying biological factors, possibly related to sex hormones or genetic differences, may influence postoperative recovery and complications independent of lifestyle factors.

With the acceleration of population aging, there is an increasing trend of elderly patients with esophageal cancer. The treatment of elderly patients is becoming an increasingly important focus of attention (16). Currently, the treatment modality for elderly patients with ESCC still centers on surgery as the cornerstone, combined with radiotherapy, chemotherapy, and immunotherapy (14, 28–31). It is important to note that the treatment of elderly ESCC patients remains a challenging clinical issue. Many patients and their families often consider non-surgical treatment options due to advanced age and comorbidities such as diabetes, hypertension, and cardiovascular diseases (16, 30, 31). While studies have shown that definitive radiotherapy or chemoradiotherapy can alleviate symptoms and prolong survival, it is crucial to recognize that non-surgical treatments do not necessarily result in fewer adverse events compared to surgical interventions. Moreover, non-surgical approaches are associated with higher tumor recurrence rates and lower overall survival outcomes (31, 32). During the period between 2016 and 2021, real-world clinical practice in our center primarily involved surgery alone as the treatment of choice for resectable tumors. This was largely influenced by several factors, including the advanced age of many patients, the frequent presence of chronic comorbidities, and preferences of patients. Although our surgeons often recommend neoadjuvant therapy combined with surgery, after thorough communication regarding treatment options, many patients and their families opted for surgery alone rather than pursuing neoadjuvant therapy followed by surgery. Over the past seven years, however, there has been a gradual increase in the proportion of patients undergoing neoadjuvant therapy, both at our center and across China, as awareness of the benefits of multimodal treatment strategies has grown.

A Japanese study demonstrated that the combination of infectious complications has an effect on survival outcomes in elderly patients (30). While elderly patients may experience more adverse events during treatment, studies have shown that neoadjuvant therapy can still lead to improved survival outcomes (6). In light of these findings, it is evident that optimizing treatment strategies for elderly ESCC patients requires a nuanced understanding of both the benefits and risks associated with different therapeutic modalities.

The higher rates of postoperative complications in male patients, as identified in our study, underscore the necessity for individualized perioperative management strategies. For instance, enhanced liver function monitoring and interventions to prevent pneumothorax might be particularly beneficial for male patients. Additionally, the role of genetic predispositions and hormonal influences on recovery and complication rates warrants further investigation and could pave the way for more personalized treatment approaches.

This study has several limitations that should be acknowledged. Firstly, as a retrospective cohort study, it is inherently subject to selection bias and residual confounding despite efforts to control for these factors through PSM. The reliance on a single-center database may limit the generalizability of the findings to other populations or healthcare settings, especially given the potential regional variations in clinical practice and patient demographics. At last, the use of administrative data may not capture all clinical nuances, such as the severity of comorbid conditions or patient-reported outcomes, which could influence survival and the incidence of postoperative complications. Future studies could address these limitations by incorporating multicenter data, extending follow-up durations, and considering broader age groups for a more comprehensive analysis.

## 5 Conclusions

This study of elderly patients aged 70 under esophagectomy found that although females exhibited a longer OS and DFS than males, the difference was not statistically significant after PSM. In patients who neither smoked nor consumed alcohol, the survival differences between sexes were similarly negligible. However, males exhibited a higher incidence of specific postoperative complications, such as abnormal liver function and pneumothorax.

## Data Availability

The raw data supporting the conclusions of this article will be made available by the authors, without undue reservation.
